# The effectiveness of a high-intensity interval exercise on cardiometabolic health and quality of life in older adults: a systematic review and meta-analysis

**DOI:** 10.1186/s13102-025-01176-5

**Published:** 2025-05-24

**Authors:** Havva Sert, Merve Gulbahar Eren, Busra Gurcay, Ferhat Koc

**Affiliations:** 1 Deparment of Internal Medicine Nursing, Faculty of Health Science, Sakarya University, Sakarya, Turkey; 2 Internal Medicine Nursing Department, Sakarya University, Institute of Health Science, Sakarya, Turkey

**Keywords:** Cardiometabolic health, High-intensity interval exercise, Meta-analysis, Older adults, Quality of life

## Abstract

**Background:**

High-intensity interval training (HIIT) is an effective exercise for improving physical and psychological function. However, there is an ongoing debate about the effects of HIIT on older adults. This study aimed to examine the effects of HIIT on cardiometabolic health and quality of life in older adults and to provide evidence-based information on the effectiveness of this type of exercise by performing a meta-analysis of randomized controlled trials.

**Methods:**

The ScienceDirect, PubMed, Web of Science, Scopus, Cochrane CENTRAL, CINAHL, and Pedro databases were used to search for all studies conducted up to December 1, 2024. Data were analyzed using Cochrane Review Manager (RevMan) [version 5.4.1] software. Quality of life data was analyzed using standardized mean difference (SMD), while mean difference (MD) was calculated for continuous variables such as heart (bpm), V̇O₂peak (mL·kg⁻^1^·min⁻^1^), 6MWT (m), and gait speed (m/s). In cases where high heterogeneity was observed, the random-effects model was preferred, and in cases where low heterogeneity was observed, the fixed-effects model was preferred.

**Results:**

This meta-analysis was performed using data from 11 studies. HIIT intervention groups had statistically significant increases in 6MWT (z = 3.24; 95% CI = [24.56, 78.67]; *p* = 0.0002), V̇O₂peak (z = 3.74; 95% CI = [1.38, 4.42]; *p* = 0.0002), and quality of life (z = 2.66; 95% CI = [0.40, 2.63]; *p* = 0.008) compared to the control groups. However, the meta-analysis indicated that HIIT resulted in non-significant changes in gait speed (z = 1.43; 95% Cl = [-0.04, 0.28]; *p* = 0.15).

**Conclusions:**

This meta-analysis revealed that HIIT interventions in older adults positively affect cardiometabolic health parameters (6MWT, V̇O₂peak) and quality of life. Conducting HIIT as a supportive treatment component with health professionals such as physicians, physiotherapists, and nurses within the framework of a multidisciplinary team approach may contribute to improving the health of older adults.

**Trial registration:**

The study was registered on the International Prospective Registry of Systematic Reviews-PROSPERO database (CRD42023481425) on November 20, 2023.

**Supplementary Information:**

The online version contains supplementary material available at 10.1186/s13102-025-01176-5.

## Introduction

Aging is a complex and inevitable process manifested by biological, psychological, and sociological changes. This process leads to structural and functional losses in the cardiovascular, musculoskeletal, and metabolic systems, affecting individuals’ general health and quality of life [1]. According to WHO data, the number of people aged 60 and over is expected to exceed 2 billion worldwide by 2050 [2]. This demographic change indicates that health problems such as cardiometabolic diseases, sarcopenia, and loss of functional independence, which are common in older people, will become more prevalent [3].

Cardiometabolic diseases increase rapidly during aging and negatively affect the quality of life [4]. Risk factors such as hypertension, diabetes, dyslipidemia, and obesity are among the leading causes of cardiovascular diseases and mortality in older adults [5]. A decrease in physical activity levels with aging accelerates the development of these diseases and increases the risk of chronic diseases [6]. Lack of exercise negatively affects cardiovascular health, muscle strength, and metabolic functions [7].

Regular physical activity significantly benefits cardiovascular health, muscle mass, metabolic function, and overall quality of life [8]. The American College of Sports Medicine (ACSM) and WHO recommend that older adults perform at least 150 min of moderate-intensity aerobic exercise or 75 min of high-intensity exercise per week [9]. However, adherence to standard exercise programs is often difficult in older people due to time constraints and lack of motivation [10].

In this context, high-intensity interval training (HIIT) has been identified as an alternative to moderate-intensity continuous exercise in cardiac rehabilitation [11]. HIIT protocols involve alternating short periods of high-intensity exercise with brief rest intervals [12]. These intense workouts performed at 80–95% of maximum heart rate and combined with low-intensity recovery periods yield effective results in a shorter time [5]. HIIT may be more effective than traditional moderate-intensity continuous training (MICT) in increasing aerobic capacity and reducing cardiometabolic risk factors [3].

Considering the literature, HIIT significantly improves cardiorespiratory fitness (V̇O₂peak), body composition, and metabolic parameters such as insulin sensitivity, glucose metabolism, and blood lipid profiles in older adults [6, 7]. In particular, HIIT programs lasting 12 weeks and longer have been reported to increase maximum oxygen consumption (V̇O₂peak), by 10–30% [13]. Furthermore, HIIT can prevent sarcopenia and improve functional independence by increasing muscle mass and strength [10]. In addition, HIIT has been reported to increase insulin sensitivity, improve glucose metabolism, and favourably affect lipid profiles [14]. However, HIIT is not only limited to physiological benefits but also positively affects quality of life. Research shows that participation in regular HIIT programs increases energy levels, facilitates activities of daily living, and significantly improves physical, psychological, and social areas [15]. Furthermore, through its modulatory effects on oxidative stress and inflammation, HIIT has been reported to reduce the risk of cardiovascular disease and support cognitive function [1, 16].

However, it is important to note that HIIT may not be suitable for all older individuals. Some studies have reported that HIIT could pose risks such as excessive cardiovascular stress, musculoskeletal injuries, or adverse events in individuals with multiple comorbidities. Therefore, careful medical screening, supervision, and program individualization are recommended before initiating HIIT in elderly populations [17].

The current literature shows significant differences in HIIT protocols’ intensity, duration, and frequency, making it difficult to compare the results [3]. Moreover, exercise modality also varies across studies (e.g., cycling, treadmill, resistance HIIT), further complicating synthesis and interpretation [18]. In addition, follow-up studies evaluating the long-term safety of HIIT and its effects outside the cardiovascular system are lacking [19]. This highlights the need for longitudinal studies to evaluate the sustainability, adherence, and broader health impacts of HIIT programs in older adults.

Given the chronic disease burden of older adults, larger-scale, well-designed studies are needed to understand the potential long-term effects of HIIT [6]. This study aimed to examine the effects of HIIT on cardiometabolic health and quality of life in older adults and to provide evidence-based information on the effectiveness of this type of exercise by meta-analyzing randomized controlled trials. Methodological heterogeneities and inconsistencies in current literature make it difficult to establish a clear framework for the effects of HIIT on the older population. This study aimed to fill these gaps and provide a more consistent analysis by evaluating different study protocols and providing findings that can guide health professionals in developing more effective and safer exercise programs for older adults.

## Methods

### Design

Studies were reported according to the Preferred Reporting Items for Systematic Reviews and Meta-Analyses (PRISMA) checklist (Supplementary File 1) [20]. Keywords were generated using the PICOS (participants, intervention, control, outcome, and study design) approach to search databases for relevant published articles (Table 1). We focused on studies with a sample of older adults aged over 60 years, regardless of their health status (P). Accordingly, the study populations comprised healthy older adults and individuals with stable chronic conditions. Eligible chronic conditions included, but were not limited to, cardiovascular diseases, chronic obstructive pulmonary disease (COPD), heart failure, hypertension, and dementia. Studies were included if participants were not experiencing acute exacerbations of their conditions and were considered clinically stable. Interventions (I) included all interventions involving high-intensity interval exercise. Patients in the control group (C) did not receive these high-intensity exercise interventions. Studies focusing on cardiometabolic health parameters and quality of life as outcomes and reporting means, standard deviations (SD), and t-scores or *p*-values were considered (O). In this systematic review, cardiometabolic health outcomes were initially defined following widely accepted criteria in the literature, including cardiorespiratory fitness measures such as peak oxygen uptake (V̇O₂peak), six-minute walk test (6MWT) performance, and gait speed and cardiovascular and metabolic parameters such as blood pressure, heart rate, lipid profile, blood glucose levels, insulin resistance indices, and body composition measurements. Randomized controlled trials (RCTs) testing the effects of high-intensity interval exercise interventions among older adults were analyzed (S). Following the PICOS approach, we addressed the research question: ‘Does high-intensity interval exercise affect cardiometabolic health and quality of life in older adults?’.

**Table 1:** The initial database search strategy following PICOS elements

Population	Intervention	Comparison intervention	Outcome measure	Study
Older people	High-intensity interval exercise interventions	No receiving any high-intensity interval exercise intervention	Cardiometabolic health and quality of life	Randomized controlled trials

The inclusion criteria were: (a) involving participants aged 60 years or older; (b) RCT design; (c) examining the effects of high-intensity interval exercise interventions; (d) written in English; and (e) moderate or high methodological quality. Exclusion criteria were (a) studies published only as abstracts; (b) studies published as observational/correlational design, case report/series, review, or non-RCT; (c) studies that were part of grey literature such as letters to the editor, theses, dissertations, conference proceedings, committee, and government reports; and (d) studies of low methodological quality.

### Search strategy

Since the comprehensive literature review was first conducted by researchers in June 2024, studies up to this date were included from inception. Then, the detailed review processes of full texts, analysis, and article writing processes were completed in December 2024. Before moving on to the journal submission process, two authors examined whether there were studies that met the inclusion criteria added to the literature between June and December 2024. Accordingly, since there were no new studies added, no changes were made. Seven electronic databases (ScienceDirect, PubMed, Web of Science, Scopus, Cochrane CENTRAL, CINAHL, and Pedro) were searched for relevant publications. Search terms included keywords, subheadings, and Medical Subject Headings (MeSH) (Table 2). Three authors (MGE, BG, and FK) performed an independent and comprehensive search of each database and applied filters based on inclusion and exclusion criteria. The International Prospective Register of Systematic Reviews (PROSPERO) ID registered for the review protocol is CRD42023481425.

**Table 2:** Comprehensive search with identified keywords

Databases (ScienceDirect, PubMed, Web of Science, Scopus, Cochrane CENTRAL, CINAHL and PEDro)				
(“older people” OR “elderly” OR “older adults” OR “old age people” OR “65 years of age or older”) AND (“high-intensity interval exercise” OR “high-intensity intermittent exercise” OR “high-intensity interval training” OR “high-intensity intermittent training”) AND (“heart rate” or “blood pressure” OR “exercise capacity “ OR “cardiometabolic fitness” OR “cardiometabolic well-being” OR “quality of life” OR “life quality” OR “health-related quality of life”) AND (“randomized controlled trial” OR “clinical trial” or “controlled clinical trials”)				
Population	Interventions	Comparison interventions	Outcome measures	Study
Older adults Elderly people Elderly Old age people 65 years of age or older	High-intensity intermittent exercise high-intensity interval traning high-intensity intermittent training	Routine care No care No any training or exercise session apart from high-intensity interval exercise interventions	Heart rate Pulse Rate Blood pressure Exercise capacity Cardiometabolic fitness Cardiometabolic well-being Quality of life Life quality Health-Related Quality of Life	Clinical trial Randomized Trial Controlled clinical trials

### Data abstraction

Mendeley Reference Manager version 2.73.0© 2022 (Elsevier, Amsterdam) was used to collect citations from the databases, and all duplicate studies were removed. Titles and abstracts were then screened for the inclusion of an RCT with a high-intensity interval exercise intervention. When full texts were not available, they were requested from the authors. After the full texts were analyzed, the reasons for excluding the studies were written on the data extraction form. Pilot testing of the data extraction form was performed to increase its reliability and validity and to improve the quality and reliability of the systematic review or meta-analysis study. Firstly, a data extraction form was prepared following the purpose and criteria of the study. This form included the main characteristics of the studies, interventions, outcomes, and other relevant information. Several studies were randomly selected among the studies to be included to test the data extraction form. Three researchers (MGE, BG, and FK) independently extracted data from the selected studies using the form. The data extracted by these researchers were compared. This process was carried out to determine whether some parts of the form were clear, what information was missing, or what parts were confusing. The other researcher (HS) checked and revised the form, considering the problems and inconsistencies from the comparison. Once it was decided that there were no problems in the pilot tests, the form was finalized and used for the main study.

The study authors, country, year of publication, sample size, participant characteristics (age and sex), inclusion criteria, disease type, intervention and control strategies, intervention providers, evaluation points, main outcome variables, and main findings of the study (mean, SD, or *p*-values and effect size) were recorded on a Microsoft Excel form with extracted data. Template for intervention description and replication (TIDieR) reporting guidelines were followed to report details of the interventions. This checklist included (1) the nature of the intervention, (2) the method and location of the intervention, (3) the number and duration of sessions, (4) the method of delivery of the intervention, and (5) the intervention areas.

### Search outcomes

For this systematic review and meta-analysis, the effects of high-intensity interval exercise on cardiometabolic health-related outcomes and the quality of life of older patients were analyzed. Outcome measures that were reported by at least three independent studies were included in the meta-analysis. Although conducting a meta-analysis with just two studies is technically possible, such analyses offer limited statistical power and restrict the accurate estimation of between-study heterogeneity [21, 22]. To ensure the robustness and reliability of the pooled effect sizes, especially when applying a random-effects model, we followed the recommendation of including a minimum of three studies per analysis [21, 22]. This approach also allowed for more meaningful interpretation of heterogeneity and increased the generalizability of the findings. In this regard, since 6MWT, V̇O₂peak, and gait speed and quality of life were evaluated as outcome parameters in at least three studies included in the systematic review, these parameters were selected for meta-analysis in the subsequent process. In addition, these measures are sensitive to changes following exercise interventions and are commonly reported in HIIT studies. Including these tools allowed for a more consistent and comparable synthesis across studies. Accordingly, the meta-analysis was performed on the following outcome variables: 6MWT (m), gait speed (m/s), V̇O₂peak (mL·kg⁻^1^·min⁻^1^), and quality of life (standardized mean scores based on self-reported questionnaires).

6MWT: It was used in four included studies [23–26] as a measure of submaximal aerobic capacity and functional exercise performance in older adults. In this test, participants were instructed to walk along a flat, straight course for six minutes at their own pace, with the total distance walked (in meters) recorded as the primary outcome. The 6MWT reflects the integrated response of the cardiovascular, respiratory, and musculoskeletal systems and is a reliable and widely used assessment of physical function in both healthy individuals and those with chronic health conditions. Higher distances indicate better functional capacity and cardiopulmonary fitness.

Gait speed: Gait speed was assessed as an indicator of lower extremity function and general physical performance in older adults across three studies [26–28]. It was typically measured by timing participants as they walked over a set distance-commonly 4 or 10 m at their usual pace. The outcome was expressed in meters per second (m/s). Gait speed is widely recognized as a reliable and sensitive marker of functional mobility in older adults, and higher gait speed values indicate better physical function and mobility.

V̇O₂peak: It was determined as the highest 20-s averaged oxygen uptake value achieved during the incremental exercise protocol, reflecting maximal exercise capacity in two studies [23, 26]. Among these studies, ventilation and pulmonary gas exchange were measured on a breath-by-breath basis using a tightly sealed face mask connected to a digital volume flow sensor and a rapid-response oxygen and carbon dioxide gas analyzer (Oxycon Pro). In another study [29], V̇O₂peak was defined as the highest 30-s average within the last minute of exercise and analyzed using cardiopulmonary exercise testing (CPET). Across these studies, VO₂peak was reported in milliliters per kilogram per minute (mL·kg⁻^1^·min⁻^1^) as the standard unit.

Quality of life: It was assessed using validated self-report instruments. Three studies [24, 27, 30]. employed the Short Form-36 Health Survey (SF-36), which evaluates eight domains of health-related quality of life, with domain scores ranging from 0 to 100, where higher scores indicate better health status. Two studies [23, 31] used the EQ-5D-3L, a standardized instrument that includes five dimensions (mobility, self-care, usual activities, pain/discomfort, and anxiety/depression), each with three severity levels, scored 0 to 1, higher scores indicate better quality of life. One study [29] utilized the Kansas City Cardiomyopathy Questionnaire (KCCQ), which is specific to patients with heart failure and assesses physical function, symptoms, social limitations, and QoL, with higher scores indicating better outcomes.

### Quality appraisal

The quality of the evidence was scored from 1 (highest) to 5 (lowest) using The Centre for Evidence-Based Medicine (CEBM) (March 2009) assessment tool [32]. The modified Jadad scale was used to assess the methodological quality of randomized controlled trials [33]. Studies with a quality score of ≥ 4 were considered good, while studies with a quality score of ≤ 3 were regarded as low quality. In this scale, 0 points represent the lowest quality, and 8 points represent the highest quality [34].

### Risk of bias

The six components of bias risk assessment were defined as selection, performance, detection, attrition, reporting, and other bias, and this assessment was performed using the Cochrane Modified Risk of Bias Tool (Version 5.4.0). Sources of risk of bias were assessed as high (-), low ( +), or uncertain (?) by three authors [35]. The other author reviewed all studies, and disagreements were resolved through team discussion. To detect publication bias, whether the effects of the studies were symmetrically distributed according to the sample size was evaluated with the help of a funnel plot. A symmetrical distribution in the funnel plot indicates no publication bias. If the symmetry is broken, the possibility of publication bias is evaluated [35, 36].

### Data synthesis

The ages of the participants in the intervention and control groups were calculated using the mean and standard deviation values given in the studies. The sex distribution of the participants was presented using percentages. Outcome parameters assessed in three or more studies, such as 6MWT (*n* = 4), V̇O₂peak (*n* = 3), gait speed (*n* = 3), and QoL (*n* = 6), were included in the meta-analysis. Of these outcomes, QoL was measured before and after the intervention using validated self-report instruments.

This meta-analysis study analyzed data using Cochrane Review Manager (RevMan) [version 5.4.1] software. Quality of life data was analyzed by standardized mean difference (SMD), while mean difference (MD) was calculated for continuous variables such as VO₂ peak, 6MWT, and gait speed. In the analysis, Higgins I^2^ statistic was used to assess the level of heterogeneity of the included studies. The I^2^ statistic was interpreted with I^2^ values of 25%, 50%, and 75% representing low, moderate, and high levels of heterogeneity, respectively. The random-effects model was preferred in cases of high heterogeneity, while the fixed-effects model was selected in cases of low heterogeneity [21]. The significance of the interventions was indicated by 95% confidence intervals (CIs), *p*-values, and Z-scores, which were used to assess the accuracy of summary estimates. All results were considered statistically significant at *p* < 0.05.

## Results

### Summary of search results

**Fig. 1:**
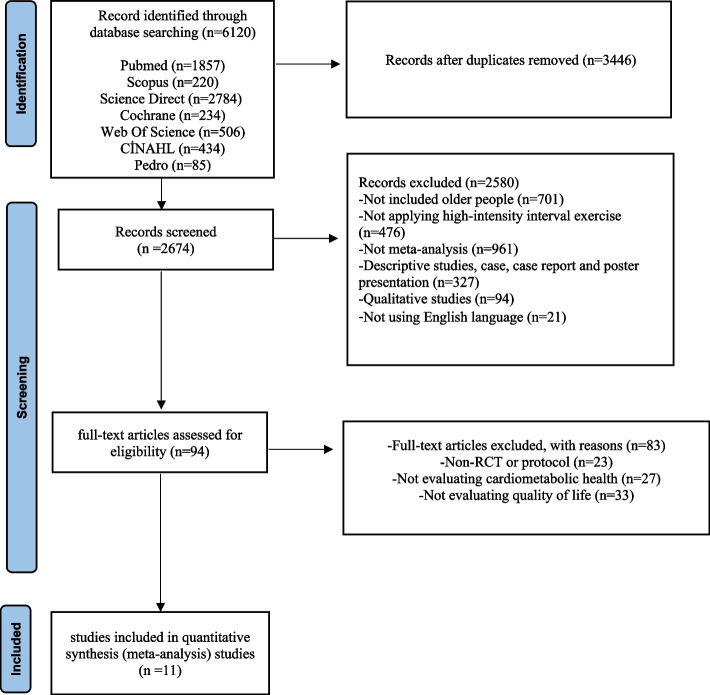
PRISMA Flow diagram. The literature search process and reasons for exclusion are summarized in the PRISMA flowchart as shown in Fig. 1. A total of 6,120 studies were reached searching seven databases. Of these studies, 3446 were excluded because they were repetitive studies. First, the remaining studies were evaluated according to the study inclusion criteria by reviewing their titles and abstracts, and 2580 studies were excluded as they did not meet the criteria. The full texts of the remaining 94 studies were reviewed according to the inclusion criteria. Subsequently, 83 studies were excluded at the full-text review stage, primarily due to the absence of a randomized controlled trial (RCT) or protocol (*n* = 23), not evaluating cardiometabolic health parameters (*n* = 27) and not evaluating

The literature review process and the reasons for exclusion are summarized in the PRISMA flowchart, as shown in Fig. 1. Seven databases were scanned, and a total of 6,120 studies were found. After duplicates were removed, 2,674 studies remained. First, the remaining studies were evaluated according to the inclusion criteria by reviewing their titles and abstracts, and 2,580 studies were omitted because they did not meet the criteria. The full texts of the remaining 94 studies were examined according to the inclusion criteria. Then, in the full-text review phase, 83 studies were excluded due to the absence of randomized controlled trials (RCTs) (*n* = 23), no assessment of cardiometabolic health parameters (*n* = 27), and no assessment of quality of life (*n* = 33). As a result, a total of 11 studies were included in this meta-analysis (Fig. 1).

### Characteristics of included studies

Eleven studies were included in this systematic review (Supplementary File 2). The studies were conducted between 2008 and 2023 and were conducted in Austria (*N* = 2) [28, 37], Spain (*N* = 4) [23, 26, 27, 30], Brazil (*N* = 1) [38], England (*N* = 1) [31], Hungary (*N* = 1) [24], Germany (*N* = 1) [29], Belgium (*N* = 1) [29] and Norway (*N* = 2) [25, 29]. One of the studies included more than one country as a multicenter study.

The studies have generally targeted adults with chronic health conditions. These health problems include cardiovascular diseases (e.g., chronic heart failure, coronary artery disease), chronic obstructive pulmonary disease (COPD), dementia, and mobility limitations [23–27, 29–31]. Participants were predominantly older people aged 66 to 90 years. The mean age was 70 years (SD = 7.3). In most studies, male participants were more common, and the proportion of males ranged between 25 and 81%. The total sample size was 1,147 people, with 689 participants in the intervention group and 458 participants in the control group.

Interventions were generally implemented for periods ranging from 4 to 12 weeks, with a frequency of 2 to 5 sessions per week, each lasting between 30 and 90 min. In most studies, HIIT protocols were applied and adapted to participants’ physical capacities. These protocols typically consisted of 3–4 min of high-intensity exercise (targeted at 80–95% of maximum heart rate), alternated with 1–3 min of active recovery performed at 40–60% of maximum heart rate [23, 24, 27, 29, 38].

Exercises were usually performed using stationary bicycles, treadmills, resistance exercise equipment, or bodyweight exercises [27, 29, 38]. In some studies, balance and strength exercises were also included in the program [23, 26].

Interventions were predominantly implemented in hospital and clinic setting [25, 31], while some studies were implemented in different settings such as community centers or participants’ homes, and especially in home-based studies, tele-rehabilitation and digital monitoring systems played an important role [27, 30]. Exercise programs were implemented mainly by physiotherapists, exercise specialists, cardiology nurses, and health personnel trained in this field [26, 29]. In some studies, participants self-monitored their exercise programs using digital devices [30].

### Risk of bias assessment

All RCTs identified in this study were categorized as level 1b evidence using the CEBM assessment tool. With the modified Jadad scale, five studies scored 5 points, one scored 6 points, and five scored 7 points, indicating good methodological quality with ≥ 4 points (Supplementary File 2).

**Fig. 2:**
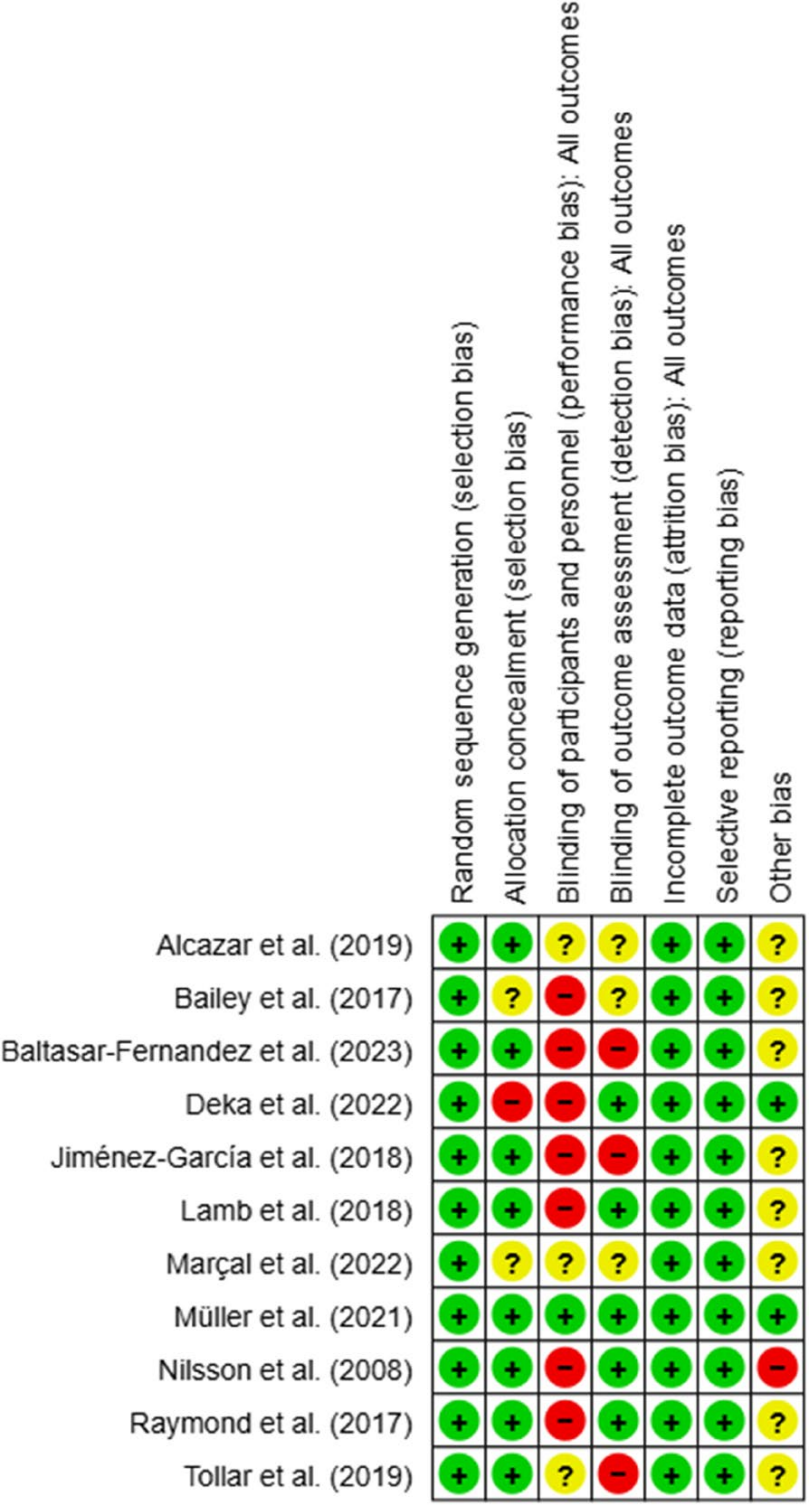
Risk of bias summary

**Fig. 3:**
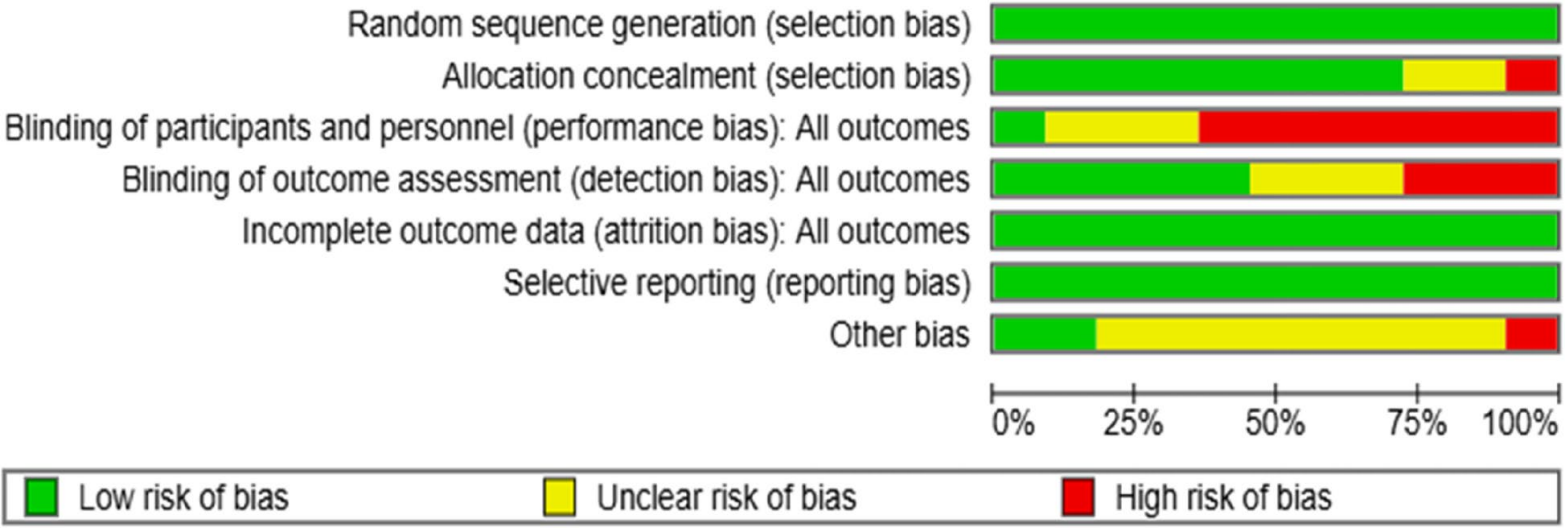
Risk of bias graph

Regarding random sequence generation, 11 RCTs (100%) showed no selection bias, while eight studies (72.7%) showed no allocation selection bias. Only one study (9%) applied participants or personnel blinding procedures for performance bias, while seven studies (63.6%) did not use blinding techniques, increasing the potential for performance bias. Similarly, only five studies (45.4%) identified outcome assessors, increasing the risk of detection bias. Attrition bias and reporting bias were found to be low risks, as the reasons for dropout and missing data were similar in the intervention and control groups in all studies, and the primary outcomes were reported. Finally, one study (9%) showed a high risk of bias regarding other risks, while 10 studies (72.7%) had an uncertain risk of other biases (Figs. 2 and 3).

### Results of the meta-analysis

This meta-analysis was conducted using data from 11 studies. This analysis explored the effects of high-intensity interval exercise interventions on outcomes related to cardiometabolic health (6MWT, gait speed, V̇O₂peak) and quality of life.

### 6MWT

**Fig. 4:**
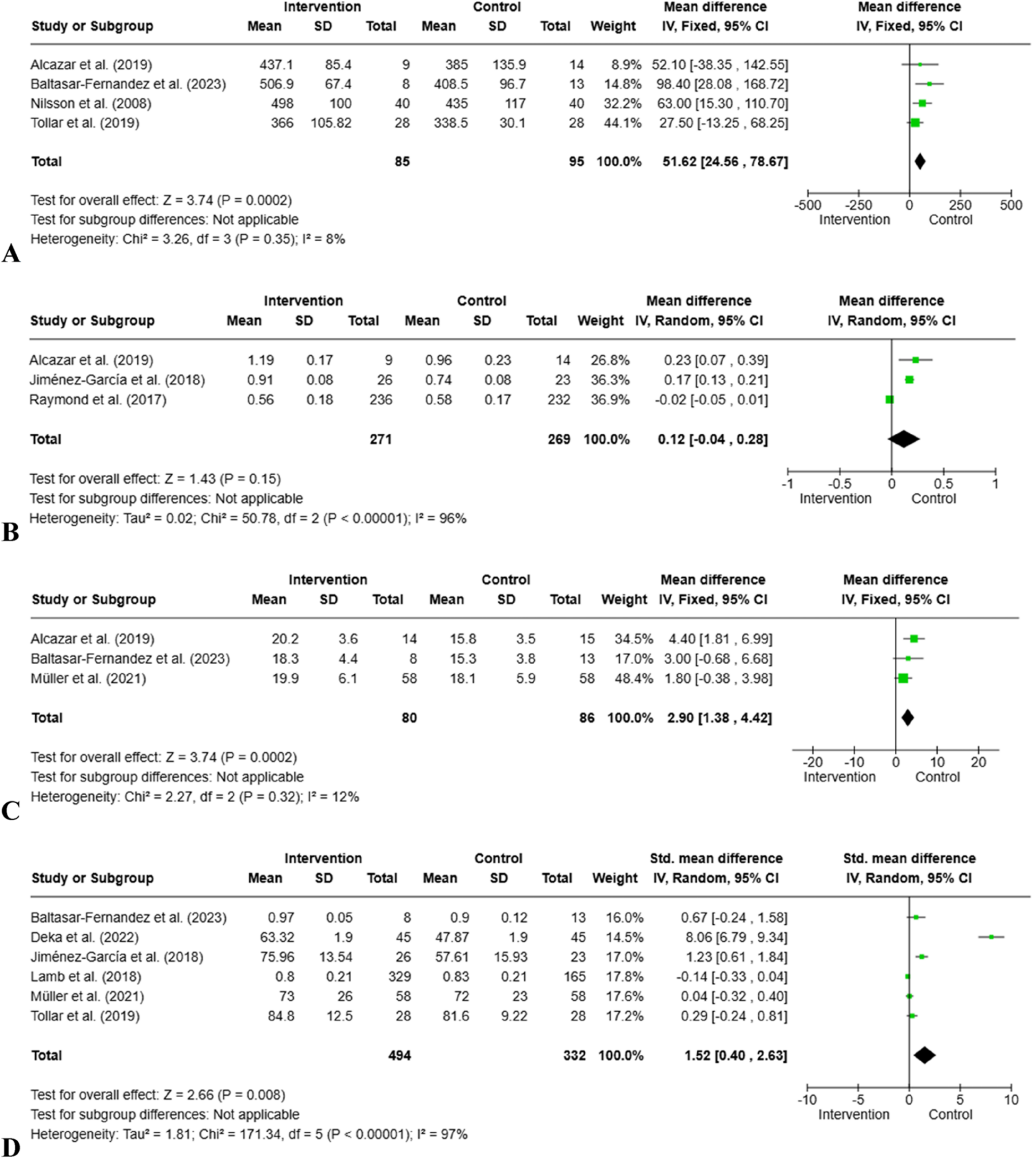
Forest Plots for cardiometabolic health and quality of life. **A**: 6MWT; **B**: Gait Speed; **C**: VO_2 Peak_, **D**: Quality of life

6MWT was measured in the four studies included in the meta-analysis. Due to the high level of homogeneity, analysis was performed using a fixed effects model (85 participants, I^2^ = 8%, Chi^2^ = 3.26, *p* = 0.35). In the intervention group, an average of 51.62 m more walking distance was measured compared to the control group. This difference was statistically significant (z = 3.24; 95% Cl = [24.56, 78.67], *p* = 0.0002) (Fig. 4).

### Gait speed

Three studies included in the meta-analysis assessed gait speed. Due to the high level of heterogeneity, they were analyzed using a random effects model (271 participants, I^2^ = 96%, Tau^2^ = 0.02, *p* < 0.00001). A slight increase in gait speed was observed in the intervention group (MD = 0.12 m/s), but this difference was not statistically significant (z = 1.43; 95% Cl = [-0.04, 0.28]; *p* = 0.15) (Fig. 4).

### V̇O₂peak

Three studies in the meta-analysis evaluated physical performance capacity with V̇O₂peak measurement. The analysis used a fixed effects model due to low levels of heterogeneity (80 participants, I2 = 12%, Chi^2^ = 2.27, *p* = 0.32). A statistically significant increase in V̇O₂peak values (MD = 2.90 ml/kg/min) was observed in the intervention group (z = 3.74; 95% Cl = [1.38, 4.42]; *p* = 0.0002) (Fig. 4).

### Quality of life

Six studies included in the meta-analysis assessed quality of life. Three studies measured QoL using the SF-36, two using the EQ-5D scale, and one using the KCCQ. Due to high levels of heterogeneity, it was analyzed using a random effects model (494 participants, I^2^ = 96%, Tau^2^ = 1.81, *p* < 0.00001). Quality of life was significantly improved (SMD = 1.52) in the intervention group compared to the control group (z = 2.66; 95% Cl = [0.40, 2.63]; *p* = 0.008) (Fig. 4).

### Publication bias assessment

**Fig. 5:**
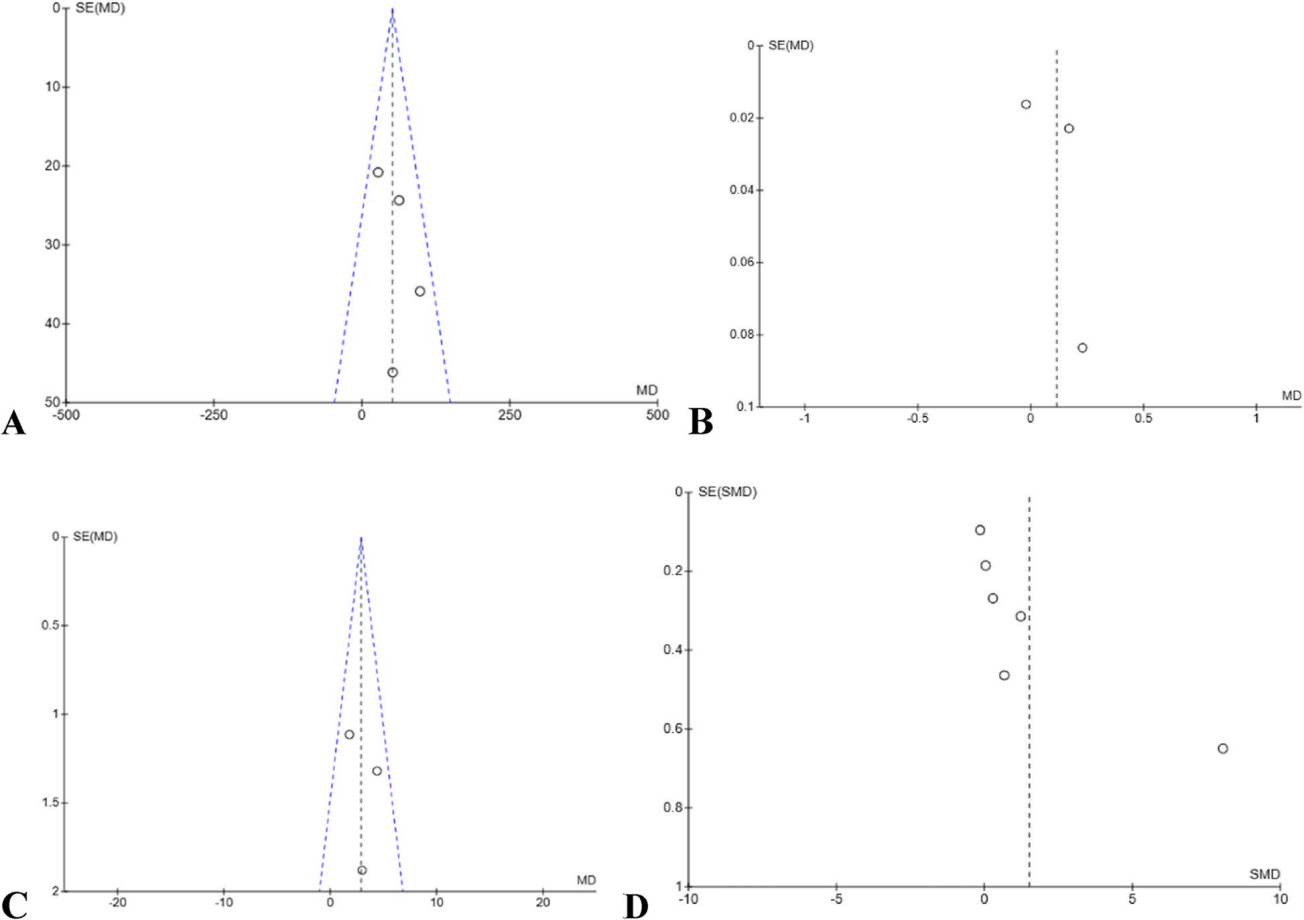
Funnel Plots for publication bias. **A**: 6MWT; **B**: Gait Speed; **C**: VO_2 Peak_; **D**: Quality of life

Publication bias was evaluated using funnel plots for each outcome with at least three included studies, in accordance with the Cochrane Handbook for Systematic Reviews of Interventions [22]. Funnel plots help visually detect potential publication bias by examining the symmetry of study effect sizes plotted against their standard errors. When the funnel plot is symmetrical, it suggests a lower risk of bias due to unpublished negative or non-significant studies. However, visual interpretation becomes less reliable when the number of studies included is fewer than 10 [39]. Accordingly, the included studies for the 6MWT and V̇O₂peak, funnel plots were found to be symmetrical, indicating a low risk of publication bias (Fig. 5). Conversely, the funnel plots for gait speed and quality of life outcomes showed marked asymmetry, raising concerns about potential publication bias (Fig. 5). Several factors could account for this, including a limited number of studies, small sample sizes, variability in intervention protocols, and selective outcome reporting [40].

## Discussion

A total of 11 randomized controlled studies were included in the meta-analysis, and it was found that HIIT had positive effects on V̇O₂peak and 6MWT, which are cardiometabolic health indicators, as a result of its application in older adults with different diagnoses. It was also concluded that HIIT improved the quality of life of older adults. Accordingly, HIIT may be evaluated as a safe and tolerable intervention applicable to different older patient populations [41–44]. When compared with the studies evaluating the effectiveness of traditional exercise interventions applied to older individuals in the literature, it is seen that studies examining the effect of HIIT intervention on cardiometabolic health parameters are limited [45–47].

### 6MWT and gait speed

Exercise is an intervention in the prevention and treatment of many diseases, such as cardiovascular diseases, diabetes, neurological diseases, cancer, obesity, and depression [48, 49]. In addition to chronic diseases, muscle strength and mass, physical activity level, and aerobic capacity decrease in old age, and individuals become dependent on daily activities. Therefore, it is necessary to increase physical activity to maintain total body mass and functional capacity in older individuals. In this direction, exercise interventions such as resistance, strength, and aerobic exercise are applied at different intensities and durations [50]. When the studies in the literature were analyzed, it was found that HIIT-treated older individuals had a more significant increase in 6MWT walking distance compared to MICT-treated older individuals [31, 42]. Baltasar-Fernandez et al. (2023) included older individuals diagnosed with COPD as an experimental group receiving HIIT and a control group receiving usual care. The study observed that the 6MWT walking distance of individuals who received the HIIT program for 12 weeks increased [23]. The findings obtained from our study show that HIIT intervention applied to older individuals increases walking distance, thus contributing positively to functional capacity. This supports the findings of literature.

In the meta-analysis study of Anjos et al. (2022) examining the effect of HIIT and aerobic exercise on cardiovascular fitness in stroke patients, it was found that individuals in the HIIT group had an increase in gait speed compared to individuals in the aerobic exercise group [51]. In a meta-analysis study by Bai et al. (2023) evaluating the effects of intensive exercise in older adults with hip fractures, it was observed that individuals who underwent intensive exercise had positive changes in gait speed, balance, and ability to perform activities of daily living [52]. In some randomized controlled trials with older adults, HIIT intervention has increased gait speed in individuals [26, 27]. Although gait speed is a widely used indicator of functional mobility and cardiovascular health in older adults, our meta-analysis found no statistically significant difference between the intervention and control groups. This result may be attributed to several factors. First, the included studies demonstrated high heterogeneity (I^2^ = 96%), which likely influenced the pooled effect size and limited the estimate’s precision. In addition, regarding publication bias, we acknowledge that the funnel plots for gait speed outcome revealed asymmetry, and the limited number of included studies (*n* = 3) further complicates the interpretation. The variability in HIIT protocols, including session duration, frequency, intensity, and mode of exercise, may have contributed to inconsistent effects on gait speed. Second, participant baseline characteristics such as functional status, comorbidities, and physical frailty could have decreased the impact of HIIT interventions. For instance, older adults with lower baseline mobility or sarcopenia may require longer intervention periods to observe measurable changes in gait speed. Additionally, gait speed is a complex outcome influenced not only by cardiometabolic capacity but also by neuromuscular and biomechanical factors, which may not be equally affected by short-term HIIT interventions [53, 54].

### V̇O₂peak

HIIT trains many different muscles simultaneously and rapidly, and glycogenolysis in the muscles occurs faster. Thus, the process of glucose uptake and glycogenesis of muscles occurs in a very short time after high-intensity interval exercises [55]. In addition, HIIT increases the production of free radicals, provides vasodilatation through nitric oxide release, and increases the expression of components such as vascular endothelial growth factor (VEGF), Insulin growth factor-1 (IGF-1), and brain-derived neurotrophic factor (BDNF) [56]. HIIT has been shown to have anti-inflammatory effects and to enhance the functions of immune cells such as neutrophils and monocytes. A review of the literature indicates that HIIT interventions influence inflammatory processes in individuals with rheumatoid arthritis, Parkinson’s disease, diabetes, obesity, metabolic syndrome, and sedentary older adults [44, 57–59]. In the study conducted by Barlett et al. (2020), which included older adults with prediabetes, a ten-week HIIT intervention resulted not only in an increase in V̇O₂peak values but also in improvements in bioenergetic neutrophil and mitochondrial functions, and thus slowed inflammation processes [44]. Additionally, HIIT has been found to regulate the levels of certain serum inflammatory markers (TNF-α, IL-10, neutrophil-to-lymphocyte ratio, neutrophil-to-monocyte ratio) in older adults with various conditions such as heart failure, obesity, and sarcopenia. Furthermore, it has been shown to positively influence mitochondrial function, body composition, and cardiometabolic fitness parameters [60, 61]. With aging, the impact of physiological changes and the presence of various comorbidities lead to an increase in oxidative stress levels in older people. Notably, cardiovascular diseases, diabetes, Alzheimer’s disease, and cancer are known to be associated with oxidative stress [62, 63]. Impairments in cardiometabolic fitness parameters affect mitochondrial biogenesis, leading to a reduction in skeletal muscle synthesis. High-intensity interval training activates the mitochondrial oxidative phosphorylation process by training different muscle groups. During HIIT, heart rate reaches 85–95% of maximum capacity, cardiac output increases, and mitochondrial production cells are activated in a cycle consisting of a balance between mitochondrial growth (biogenesis) and degradation (mitophagy) [11, 64]. In this context, HIIT is an exercise intervention that significantly induces biogenesis and promotes remodeling [65]. Studies conducted on adult individuals with different disease groups have shown that HIIT regulates key mitochondrial enzymes such as citrate synthase (CS), PGC-1α, beta-hydroxyacyl-CoA dehydrogenase (β-HAD), and cytochrome C (COX-IV), while also enhancing mitochondrial protein synthesis and biogenesis [26, 66–69].

In the meta-analysis conducted by Oliveira et al. (2024) evaluating the effects of HIIT and MICT on physical fitness in older adults, an increase in V̇O₂peak values, a key cardiometabolic fitness parameter, was observed only in individuals who underwent HIIT [70]. In a meta-analysis study by Bouaziz et al. (2020) evaluating the effectiveness of HIIT and traditional endurance training in individuals aged 65 years and older, it was determined that individuals in the HIIT group had a greater improvement in V̇O₂peak value [71]. In a meta-analysis study by Leal et al. (2020) examining the effectiveness of HIIT versus MICT in hypertensive patients, it was found that HIIT intervention provided more favorable changes in diastolic blood pressure and V̇O₂peak values [72]. In the meta-analysis study by Wu et al. (2021) evaluating the effect of HIIT intervention on physical fitness, metabolic parameters, and cardiovascular fitness in older adults, it was determined that there was a greater increase in V̇O₂peak value compared to MICT [3]. In a meta-analysis study by Yue et al. (2022) involving individuals with cardiovascular disease and receiving cardiac rehabilitation, HIIT improved cardiorespiratory fitness parameters more than MICT [73]. In meta-analysis studies in which different patient groups were included in the literature and the effect of HIIT on V̇O₂peak value was examined, it was determined that there was an improvement in V̇O₂peak value and cardiometabolic parameters of individuals who applied HIIT [3, 74]. In addition, randomized controlled trials in the literature, including older individuals with different disease diagnoses, found that HIIT intervention provided a greater increase in peak VO_2_ compared to MICT [15, 25, 26, 49, 75–82]. On the other hand, in the study by Mueller et al. (2021) involving older individuals diagnosed with heart failure with preserved ejection fraction, no difference was found in the effect of HIIT and MICT interventions on V̇O₂peak value [29]. Our findings, consistent with the literature, show that HIIT is an alternative, effective exercise intervention to increase V̇O₂peak value evaluated within the scope of cardiorespiratory fitness by increasing aerobic capacity in older individuals.

### Quality of life

Quality of life is a concept that encompasses physical, psychological, social, emotional, economic, and cultural dimensions and expresses the individual’s perception of their situation in life in terms of their goals, expectations, and concerns [83]. Exercise is essential to quality of life, and regular physical activity is vital to protect the older person’s physical and mental health. Different exercise interventions applied to older individuals help to increase their functionality and independence in daily activities, reduce the likelihood of geriatric syndromes, and protect their psychological well-being [42, 82, 84]. Baltasar-Fernandez et al. (2023) included 21 older individuals diagnosed with COPD, the patients were given HIIT for 12 weeks and it was found that the quality of life of the individuals in the intervention group was higher [23]. A meta-analysis study by Herranz-Gómez et al. (2022) revealed that HIIT intervention had a positive effect on cardiorespiratory fitness and quality of life in individuals with cancer or cancer survivors [85]. The study by Deka et al. (2022) included elderly individuals with coronary artery disease; It was divided into two groups: HIIT + resistance exercise and control group [30]. The physical capacity and quality of life of the older in the intervention group were found to be higher than the control group. In randomized controlled trials in which older individuals with different diagnoses were included, it was determined that the quality of life of individuals with HIIT increased [27, 86–88]. On the other hand, Reed et al. (2022) included 94 patients with atrial fibrillation and examined the effectiveness of HIIT and MICT programs [42]. It was determined that there was no significant increase in disease-specific and general quality of life in both intervention groups. Our study findings are consistent with the literature, and high-intensity interval exercise positively affects quality of life variables in which psychosocial status is evaluated in addition to physical parameters in older individuals. On the other hand, the important point to remember is that there is heterogeneity among the studies included in this meta-analysis. The first factor that causes this heterogeneity is sample characteristics. The studies included in our meta-analysis included patients with different comorbidities such as COPD, diabetes, coronary artery disease, heart failure, dementia, and healthy older adults. Patients with different medical diagnoses may change the effects of HIIT. The second factor is the difference in intervention protocols. HIIT applied to patients with different durations, frequencies, and protocols may be a factor that changes the size of the observed effects. The scales used to evaluate the parameters we examined in the studies were diverse. This may have led to differences in effect sizes.

### Limitations

There are some limitations in this meta-analysis. Firstly, since most of the RCTs were conducted in European countries, there may be ethnic, geographical, and cultural differences in the effectiveness of HIIT in older people and the qualifications of the interventionists. Furthermore, most studies included in this meta-analysis included individuals with chronic diseases such as COPD, heart failure, hypertension, and dementia. Therefore, RCTs, including HIIT interventions, must investigate the effect on holistic health outcomes in individuals with other chronic diseases or healthy older individuals from different cultures. These two critical points may limit the generalizability of the findings to all older individuals. Another limitation of our study may be that the scans were conducted only in English. Although the overall methodological quality of RCTs is good, it is essential to remember that some of the studies have methodological weaknesses related to small sample sizes, lack of blinding procedures, and failure to report side effects of interventions. Therefore, the evidence for the impact of methodologically well-planned RCTs with HIIT interventions in older adults using blinding procedures, large sample groups, and long-term follow-up periods should be strengthened. Additionally, the HIIT protocols varied considerably across the included studies regarding intensity, duration, frequency, and recovery periods. This heterogeneity may have influenced the pooled outcomes and complicates the ability to draw standardized conclusions about the optimal HIIT regimen for older adults. Finally, another limitation of the study was that the quality of life meta-analyzed results were based on patient subjective reports, which may introduce the potential for recall bias. Accordingly, participants’ self-assessments may have been influenced by memory distortions, current mood, or expectations about the intervention, all of which can affect the accuracy of reported quality of life scores in included studies. This limitation may reduce the findings’ reliability and internal validity and should be considered when interpreting the overall effects of HIIT on quality of life in older adults.

## Conclusion

A total of 11 randomized controlled trials were included in this meta-analysis and revealed that high-intensity interval exercise applied to older people positively affected cardiometabolic health parameters (6MWT, V̇O₂peak) and quality of life. It was found that HIIT increased 6MWT walking distance and V̇O₂peak but did not affect gait speed. The findings of our study show that in addition to the positive effects of high-intensity interval exercise on physical parameters, it also contributes to the overall quality of life, including psychological, social, and emotional aspects of individuals. Encouraging healthy and older adults with different comorbidities to exercise and including exercise as a supportive treatment component in care services can improve their physical and mental health. Carrying out exercise practices with health professionals such as physicians, physiotherapists, and nurses within the framework of a multidisciplinary team approach will help to evaluate older adults with a holistic approach. The inclusion of HIIT intervention, which is an easily applicable, non-invasive, low cost, and low side effect type of exercise, in the daily life activities of older individuals, may provide more permanent improvements in the long term. Future research should focus on conducting large-scale, long-term RCTs to evaluate HIIT interventions’ safety, sustainability, and effectiveness in diverse populations of older adults, including those from different cultural and health backgrounds. Such studies would contribute significantly to developing more inclusive and evidence-based exercise recommendations to ensure the broader applicability of HIIT in geriatric care.

## Supplementary Information

Supplementary Material 1.

Supplementary Material 2.

## Data Availability

Data sharing is not applicable to this article as no datasets were generated or analyzed during the current study.
