# Supplementary material for: The effectiveness of a high-intensity interval exercise on cardiometabolic health and quality of life in older adults: a systematic review and meta-analysis
Source: BMC Sports Sci Med Rehabil. 2025 May 24;17:128. doi: 10.1186/s13102-025-01176-5 (PMC12102952; doi:10.1186/s13102-025-01176-5)
Supplement: Supplementary file 2 — Supplementary Material 2. [file 13102_2025_1176_MOESM2_ESM.docx]

**Supplementary File 1.** Characteristics of reviewed studies

| Study authors  (Country) | Sample | Inclusion criteria | Type of Disease | Intervention strategies | Control strategies | Time points of assessment | Main variables | Study outcomes | Quality Score*/  Evidence level** |
| --- | --- | --- | --- | --- | --- | --- | --- | --- | --- |
| Lamb et al. (2018)  (England) | Intervention group  Moderate to high-intensity  aerobic and strength exercise training (n=329) or Control group (n=165)  **Age:**  Mean= 77  years  SD= 7.9 years  **Female:** 39%  **Male:** 61% | -People with dementia were eligible if they had  a clinically confirmed diagnosis of dementia in  accordance with the  DSM-IV  -A sMMSE of greater than 10  -Being able to sit on a chair  and walk 10 feet (3.05 m) without assistance  -Living in the community either alone or with others. | Dementia | The intervention group attended group sessions in a gym twice a week for four months, lasting 60 to 90 minutes. In each group session, aerobic exercise consisted of static cycling with a five-minute warm-up period followed by up to 25 minutes of moderate to hard intensity cycling, depending on tolerance level. We also asked the participants to do home exercises for one additional hour each week during this period. The supervised program lasted four months, after which we prescribed a more frequent  home-based program with a target of unsupervised  physical activity or exercise of 150 minutes each week  (total). | All participants received the usual care by clinical guidance that included counseling  for carers and families, a clinical assessment,  prescription of symptomatic treatments, and brief  advice about physical activity. The participants’  doctors determined additional therapy on the basis  of clinical need. | Baseline  6 months  12 months | -ADAScog  -Neuropsychiatric  index  -BADL  -EQ-5D  -The QoL Alzheimer’s disease scale  -Zarit burden interview  -6MWT | -By 12 months, the mean ADAS-cog score had increased in the exercise and usual care arms.  -No differences were found in secondary outcomes or preplanned subgroup analyses by dementia type (Alzheimer’s disease or other), severity of cognitive impairment, sex, and mobility.  -Six-minute walking distance improved over six weeks. | 7 (good)/1b |
| Tollar et al. (2019)  (Hungary) | Intervention groups:  EXE  (n = 28) and CYC (n = 27) or  Control group (n=28)  **Age:**  Mean= 69.6  years  SD= 3.53 years  **Female:** 53%  **Male:** 47% | - Being older  than 60 years,  -Hospital referral from the family physician for a  specific medical condition, including mobility difficulty | Mobility-limited older adults | -Each 60-minute EXE session started with a 5-minute warm-up, followed by 45 min of EXE or CYC, concluded with a 5-minute cool-down, and included 5 min of rest as needed during the 45-minute segments. Warm-up consisted of walking on toes and heels, inside and outside of the sole, with and without hand-held apparatuses (rods with small, weighted balls at the end, jogging, and stretching in sitting, lying, and standing.  -In a “spinning class,” CYC participants rode a bicycle ergometer at target HR, received no visual feedback, and listened to music. Five-minute bouts were interspersed with 1 minute of freewheeling to improve cardiovascular fitness. | Waitlisted controls did not receive any exercise. | -Baseline  -5 weeks | -SF-36  -Osteoarthritis Index  -SE-ADL  -6MWT  -BESTest  -DGI  -HR  -RPE  -BP  -MAP | -Health-related quality of life, mobility limitation, body mass, depression, and walking capacity improved similarly after the two interventions.  -Activities of daily living, Berg Balance Score, BestTest scores, and Dynamic Gait Index improved more after EXE than CYC.  -The center of pressure on the standing sway path improved in one of six tests only after EXE.  -Postexercise cardiovascular response improved in EXE.  -CON did not change in any outcomes. | 5 (good)/1b |
| Raymond et al.  (2017) | Control group (n=232)  or  Intervention group (n=236)  **Age:**  Mean= 84  years  SD=7- years  **Female:** 60,47%  **Male:** 39,53% | - Adults aged ≥65.  - Physically able to participate in weight-bearing exercise.  -Able to stand from a chair with minimum or no assistance. | Fracture | -Patients participated in group exercises three times a week in six groups.  - Additionally, patients did individual exercises once a week.  -Group sessions lasted between 45-60 minutes.  -Exercises included lower limb progressive resistance strength exercises in both supported (i.e., holding onto rails) and unsupported positions.  -Balance exercises were performed to challenge postural stability.  -Each exercise was performed for 8-12 repetitions.  **The intervention providers:** Not Given | The control group received no intervention | -Information not available | -Elderly Mobility Scale  -Berg Balance Scale  - 6-min Walk Test | A high-intensity exercise group combined with individual physiotherapy may be an effective and efficient method to provide care to older inpatients | 7 |
| Jiménez-García et al.  (2018) | Control group (n=23)  or  MIIT group (n=24)  or  HIIT group (n:26)  **Age:** Information not available  **Female:** Information not available | - Adults aged ≥60  -Able to understand and follow exercise instructions | Information not available | MIIT Group:  -Main squat activity: 4-minute intervals at 90–95% of maximum heart rate (HR) with 3-minute active rest intervals at 50–70% of maximum HR (25 minutes)  - MIIT sessions took place on Monday and Wednesday, always at the same time (10:00–11:00 a.m.), providing a 24-hour rest between sessions.  HIIT Group:  - Main squat activity: 4-minute intervals at 90–95% of maximum heart rate (HR) with 3-minute active rest intervals at 50–70% of maximum HR (25 minutes)  -HIIT sessions took place on Tuesday and Thursday, always at the same time (10:00–11:00 a.m.), providing a 24-hour rest between sessions. | Control Group: -They maintained their daily lifestyle and received a series of guidelines to encourage physical activity but were instructed to refrain from participating in any systematized exercise activity. | Exercise sessions were conducted twice a week for 12 weeks | -Anthropometry  - Body composition  - Handgrip strength  - Timed Up-and-Go test  - Short-Form -36 | This study revealed that a HIIT program with suspension exercises significantly improved gait speed, handgrip strength, BMI, and health-related quality of life in adults aged 60 and over. The HIIT group showed more significant progress in these parameters than the MIIT group. The results indicate that the HIIT program is an effective and economical method for improving the health of older adults. | 5 |
| Baltazar-Fernandez et al.  (2023) | Control group (n=13)  or  Intervention group (n=8)  **Age**: 76,9  SD: 6.8  **Female:** 19,5%  **Male:** 80.5% | -Being 65 years old or older  - Diagnosed with COPD by a pulmonologist  -Being clinically stable | COPD | Intervention Group:  - Each session started with a warm-up on a cycle ergometer, followed by power-oriented resistance training (RT) on leg press and chest press machines, and high-intensity interval training (HIIT) on a cycle ergometer. The power-oriented RT consisted of three sets of 8 repetitions at maximum power for each exercise, while the HIIT consisted of 6 to 10 sets of 90 seconds at 40%-45% Wpeak and 30 seconds at 80%-90% Wpeak. Loads were adjusted every 4 weeks and in the sixth week based on individual assessments. Two sports scientists supervised the sessions, and verbal encouragement was provided to participants. Participants were required to attend at least 80% of the sessions. | Control Group: Standard care was applied. | Early assessment was conducted at 12 weeks, and late evaluation was conducted at 10 months | -Short Physical Performance Battery (SPPB)  -Health-Related Quality of Life (EQ-5D-5L)  -Vastus Lateralis Muscle Thickness (MT)  -Peak Pulmonary Oxygen Uptake (peak VO2)  -Peak Work Rate (Wpeak)  -Early and Late Isometric Rate of Force Development (RFD)  -Leg and Chest Press Maximum Muscle Power (LPmax and CPmax)  -Systemic Oxidative Damage and Antioxidant Capacity | Twelve weeks of concurrent training improved physical function, health-related quality of life, early isometric rate of force development, maximum muscle power in older adults with COPD, and preserved muscle thickness and maximum work rate. However, this training did not have a lasting effect on peak pulmonary oxygen uptake, late isometric rate of force development, systemic oxidative damage, and antioxidant capacity after 10 months of detraining | 5 |
| Roque Marçal et al.  (2022) | Control group (n=20)  or  MICE group (n=20)  Or  HIIE Group (n=20)  **Age**: 67  SD: 7  **Female:** 45%  **Male:** 55% | -Age ≥ 60 years.  -Hypertension diagnosis: Participants must have been diagnosed with stage 1 or 2 hypertension for at least 6 months.  -No change in antihypertensive treatment: There should be no changes in antihypertensive treatment in the last 3 months.  -Office Blood Pressure: The blood pressure must be below 140/90 mmHg.  -No involvement in any structured exercise program or being physically inactive: Moderate- or vigorous-intensity physical activity levels must be less than 150 minutes or 75 minutes per week, respectively (assessed through the International Physical Activity Questionnaire). | Hypertension | Participants were divided into three groups; however, each participant participated in all groups and received each intervention in sequence.  -HIIE (High-Intensity Interval Exercise): A high-intensity interval exercise session conducted in heated water.  -MICE (Moderate-Intensity Continuous Exercise): A moderate-intensity continuous exercise session conducted in heated water. | CON (Control): A control session where no exercise was performed. | -PRE (Before): Measurements taken before the intervention, i.e., before participants entered the pool.  -POST (Immediately After): Measurements taken immediately after the intervention.  -REC (45 Minutes After): Measurements taken 45 minutes after the intervention. | -Blood Pressure (BP)  -Arterial Stiffness  -Endothelial Reactivity  -Heart Rate Variability (HRV) | According to the study results, high-intensity interval exercise (HIIE) conducted in heated water was superior to moderate-intensity continuous exercise (MICE) in improving short-term parasympathetic modulation in older individuals with hypertension. However, both types of exercise negatively affected long-term cardiac autonomic response and did not achieve the expected positive hemodynamic and vascular improvements, such as post-exercise hypotension | 6 |
| Deka et al. (2022)  USA | Intervention group (n=45)  Age: 69.20± 4.18  Female: 9 (20)  Male: 36 (80)  Control group (n=45)  Age: 69.27±5.68  Female: 13 (28.89)  Male: 32 (71.11) | Participants included in the study were older than 60 years with a diagnosis of CAD with or without invasive intervention, with sinus rhythm, preserved left ventricular function, and clinically stable. | Coronary arterial diseases | The Experimental Group participants received eight treatment sessions, one session/week that lasted 50-60 minutes. | Participants in the Usual Care group received conventional medical treatment based on the pharmacologic treatment of the disease and the relief of clinical symptoms. Standard medications for all participants were stabilized before enrollment in the study. Participants were on this medication for a minimum of 4 weeks. | -Baseline  -After 8 weeks (post-intervention) | -International Physical Activity Questionnaire)  -Incremental Shuttle Walking  -Blood pressure  -Heart rate  -SF-36 | Significant group and time interaction were found for the participants in the HIIT þ R Group for BMI (P 1⁄4 .001), body fat percentage, waist circumference, physical activity, functional capacity, and QoL compared with the UC Group. Significant improvement in systolic blood pressure was seen in the HIIT þ R group. | 7 |
| Nilsson et al. (2008)  Norway | Intervention group (n=40)  Age: 68.8±7.9  Female: 9 (%22.5)  Male: 31 (77.5)  Control group (n=40)  Age: 71.5±7.8  Female: 8 (%20)  Male: 32 (%80) | All patients referred to our heart failure outpatient clinic were screened for inclusion in this study. | Heart failure | The exercise program consisted of group-based simple aerobic dance movements (with music) 2 days a week for 4 months. Each session lasted 50 minutes (including warm-up and cool-down), followed by 15 to 30 minutes of counseling. | The control group was not provided with exercise prescriptions. They were encouraged to continue their usual physical activity and were not discouraged from regular physical activity. | -Baseline assessment  - 4 months follow up  -12 months follow up | -6MWT  -Minnesota Living With Heart Failure Questionnaire score | After 4 months, functional capacity (6-minute walk distance 58 vs. 15 m) and quality of life (Minnesota Living with Heart Failure Questionnaire score 10 vs. 1 point) improved significantly in the exercise group compared with the control group. After 12 months, improvements in all parameters were significant in the exercise group compared with the control group. In conclusion, the results support implementing a group-based aerobic interval training program to improve the long-term effects on functional capacity and quality of life in patients with CHF. | 7 |
| Alcazar et al. (2019)  Spain | Intervention group (n=14)  Age: 77.7 ± 7.9  Female: 3  Male: 11  Control group (n=15)  Age: 79.8 ± 6.4  Female: 2  Male: 13 | Before entering the study, the subjects had to be ≥65 years old, diagnosed with COPD by a pulmonologist, and clinically stable. | COPD | The participants assigned to the ET group attended 24 exercise training sessions on non‐consecutive days during 12 weeks (i.e., 2 days a week). Each session consisted of a 5‐minute warm‐up on the cycle ergometer (60‐80 rpm; 40% of Wpeak). This was followed by power training in the leg and chest press exercise machines and endurance HIIT on the cycle ergometer. | CT group received no intervention (i.e., usual care) during the same period. | -Baseline  -After 12 weeks | -6MWT  -Cardiopulmonary outcomes  -C  -Resting SpO2  -FEV1/FVC (%)  -VO_2_ peak | The combination of HIIT and power training improved systemic oxidative stress and limb muscle dysfunction in older people with COPD. Changes in oxidative stress were associated with exercise‐induced structural and functional adaptations. | 5 |
| Bailey et al. (2017)  (Australia) | Intervention groups:  Lower CRF (n=27)  Higher CRF (n=20)  **Age:**  Mean= 70.33  years  SD= 5 years  **Female:** 0%  **Male:** 100% | -Aged 70 ±5 years  -BMI: 25.3±3.4 kg/m^2^  ^-^Being able to exercise regarding a pre-exercise screening questionnaire  -Being nonsmokers (>12 months no smoking history). | Healthy elderly males | Both acute exercise protocols commenced with a 3-min warm-up at 0 W, followed by either 24 min of *1*) continuous moderate-intensity cycling at 40% peak power output or *2*) high-intensity interval cycling involving twelve 60-s bouts at 70% peak power output, with each separated by 60 s at 10% peak power output. | The control consisted of 27 min of seated rest with both arms relaxed and rested on a table in front. | -Baseline  -Heart rate and RPE were recorded every 2 min.  - FMD measurements at 10 and 60 min.  -Right brachial artery blood pressure was measured in triplicate using an automated device 10 min before each FMD time point. | -Heart rate  - RPE using the 0–10 Borg scale  -MAP  -SBP  -DBP  -Endothelial function using Brachial artery FMD parameters [brachial diameter, peak diameter, and FMD (mm),  FMD (%), time to peak, shear rate area under the curve (SR_AUC_), and  blood flow] | -FMD increased after MICE in both groups and normalized after 60 min.  -In the lower fit group, FMD was reduced after HIIE and decreased at 60 min.  -In the higher fit group, FMD was unchanged immediately after HIIE and increased after 60 min.  -In the no-exercise control, FMD was reduced in both groups after 60 min. | 5 (good)/1b |
| Mueller et al. (2021), Germany | -HIIT (n = 58)  Age: 70±7  Female: 41±71  -MCT (n = 58)  Age: 70±8  Female: 35±60  -Control group (n=60)  Age: 69±10  Female: 41±68 | Sedentary patients with signs and symptoms of HFpEF (exertional dyspnea [New York Heart Association class II-III], LVEF of 50% or more significant, and elevated estimated LV filling pressure [E/e′ medial ≥15] or E/e′ medial of 8 or greater with concurrent elevated natriuretic peptides [NT-proBNP ≥220 pg/mL or BNP ≥80 pg/mL])14 were eligible to participate in the trial. | Heart failure | High-intensity interval training was scheduled 3 times per week for 38 minutes per session  Moderate continuous training was scheduled 5 times per week for 40 minutes per session | Patients assigned to guideline control received 1-time advice on physical activity according to guidelines. | Baseline and 3, 6, and 12 months | -Anthropometry, electrocardiogram, blood analysis, cardiopulmonary exercise testing, echocardiography,  -KCCQ  -VO2peak  -NT-proBNP | Among patients with HFpEF, there was no statistically significant difference in change in peak VO2 at 3 months between those assigned to high-intensity interval vs moderate continuous training, and neither group met the prespecified minimal clinically significant difference compared with the guideline control. These findings do not support high-intensity interval or moderate continuous training compared with guideline-based physical activity for patients with HFpEF. | 8 |

*Abbreviations:* ADAScog: Alzheimer disease assessment scale cognitive subscale, AF: Atrial Fibrillation, BESTest: Balance Evaluation Systems Test, CR: Cardiovascular rehabilitation, CRF: Cardiorespiratory fitness, BMI: Body mass index, CYC: Stationary cycling, DBP: Diastolic blood pressure, DGI: Dynamic Gait Index HbA1C: Hemoglobin A1C, HDL: High-density lipoprotein, EXE: High-intensity agility exergaming, HRpeak: Peak heart rate, HRrest: Resting heart rate, HOMA-IR: Homeostasis model assessment of insulin resistance, HIIE: High-intensity interval exercise, FMD: Flow-mediated dilation, LDL: Low-density lipoprotein, KCCQ: Kansas City Cardiomyopathy Questionnaire, MAP: Mean arterial pressure, MICE: Moderate-intensity continuous exercise, RPE: Rate of perceived exertion, SBP: Systolic blood pressure, VO2peak: Peak oxygen consumption, SF-36: The Short Form-36-Health Survey, SE-ADL: Schwab-England Activities of Daily Living scale, T2DM: Type 2 diabetes mellitus, TST: Traditional strength training, PT: Power training, QoL: Quality of Life
